# Synthesis, characterization, and antibacterial activities of a heteroscorpionate derivative platinum complex against methicillin-resistant *Staphylococcus aureus*

**DOI:** 10.3389/fcimb.2023.1100947

**Published:** 2023-03-27

**Authors:** Syong H. Nam-Cha, Elena Domínguez-Jurado, Selena L. Tinoco-Valencia, Ramón Pérez-Tanoira, Noelia Morata-Moreno, Rocío Alfaro-Ruiza, Agustín Lara-Sánchez, Jaime Esteban, Rafael Luján, Carlos Alonso-Moreno, Pedro Seguí, Alberto Ocaña, Ángel López Gónzalez, John J. Aguilera-Correa, Francisco C. Pérez-Martínez, Milagros Molina Alarcón

**Affiliations:** ^1^ Department of Pathology, Complejo Hospitalario Universitario, Albacete, Spain; ^2^ Departamento de Química Inorgánica, Orgánica y Bioquímica-Centro de Innovación en Química Avanzada (ORFEO-CINQA), Facultad de Farmacia, Universidad de Castilla-La Mancha, Albacete, Spain; ^3^ Unidad nanoDrug, Centro Regional de Investigación Biomédicas, Universidad de Castilla-La Mancha, Albacete, Spain; ^4^ Clinical Microbiology Department, IIS-Fundacion Jimenez Diaz-UAM, Madrid, Spain; ^5^ Clinical Microbiology Department, Hospital Universitario Príncipe de Asturias, Madrid, Spain; ^6^ Biomedicine y Biotechnology Department, School of Medicine, University of Alcalá de Henares, Alcalá de Henares, Spain; ^7^ Department of Otorrinolaringology, Complejo Hospitalario Universitario, Albacete, Spain; ^8^ Instituto de Investigación en Discapacidades Neurológicas (IDINE), University of Castilla-La Mancha, Albacete, Spain; ^9^ CIBER de Enfermedades Infecciosas (CIBERINFEC), Instituto de Salud Carlos III, Madrid, Spain; ^10^ Experimental Therapeutics Unit, Hospital Clínico San Carlos, IdISSC and CIBERONC, Madrid, Spain; ^11^ Translational Research Unit, Albacete University Hospital, Albacete, Spain; ^12^ Department of Nursing, University of Castilla-La Mancha, Albacete, Spain

**Keywords:** Staphycoccus aureus, metallodrug, platinum, MRSA, biofilm

## Abstract

*Staphylococcus aureus* is one of the species with the greatest clinical importance and greatest impact on public health. In fact, methicillin-resistant *S. aureus* (MRSA) is considered a pandemic pathogen, being essential to develop effective medicines and combat its rapid spread. This study aimed to foster the translation of clinical research outcomes based on metallodrugs into clinical practice for the treatment of MRSA. Bearing in mind the promising anti-Gram-positive effect of the heteroscorpionate ligand 1,1’-(2-(4-isopropylphenyl)ethane-1,1-diyl)bis(3,5-dimethyl-1H-pyrazole) (2P), we propose the coordination of this compound to platinum as a clinical strategy with the ultimate aim of overcoming resistance in the treatment of MRSA. Therefore, the novel metallodrug 2P-Pt were synthetized, fully characterized and its antibacterial effect against the planktonic and biofilm state of *S. aureus* evaluated. In this sense, three different strains of *S. aureus* were studied, one collection strain of *S. aureus* sensitive to methicillin and two clinical MRSA strains. To appraise the antibacterial activity, minimum inhibitory concentration (MIC), minimum bactericidal concentration (MBC), minimum biofilm inhibitory concentration (MBIC), and minimum biofilm eradication concentration (MBEC) were determined. Moreover, successful outcomes on the development of biofilm in a wound-like medium were obtained. The mechanism of action for 2P-Pt was proposed by measuring the MIC and MBC with EDTA (cation mediated mechanism) and DMSO (exogenous oxidative stress mechanism). Moreover, to shed light on the plausible antistaphylococcal mechanism of this novel platinum agent, additional experiments using transmission electron microscopy were carried out. 2P-Pt inhibited the growth and eradicated the three strains evaluated in the planktonic state. Another point worth stressing is the inhibition in the growth of MRSA biofilm even in a wounded medium. The results of this work support this novel agent as a promising therapeutic alternative for preventing infections caused by MRSA.

## Introduction

*Staphylococcus aureus* exists in a polymicrobial environment, primarily as a human commensal organism. However, this bacterial species can frequently cause disease in immunocompromised patients or in those that are frequently exposed to catheter insertions or injections (Frost et al., 2019). In this context, in western countries, *S. aureus* is responsible of up to 76% of skin and soft tissue infections, causing approximately 500,000 hospital visits and more than ten million outpatient visits per year (Parlet et al., 2019).

**Figure 1:**
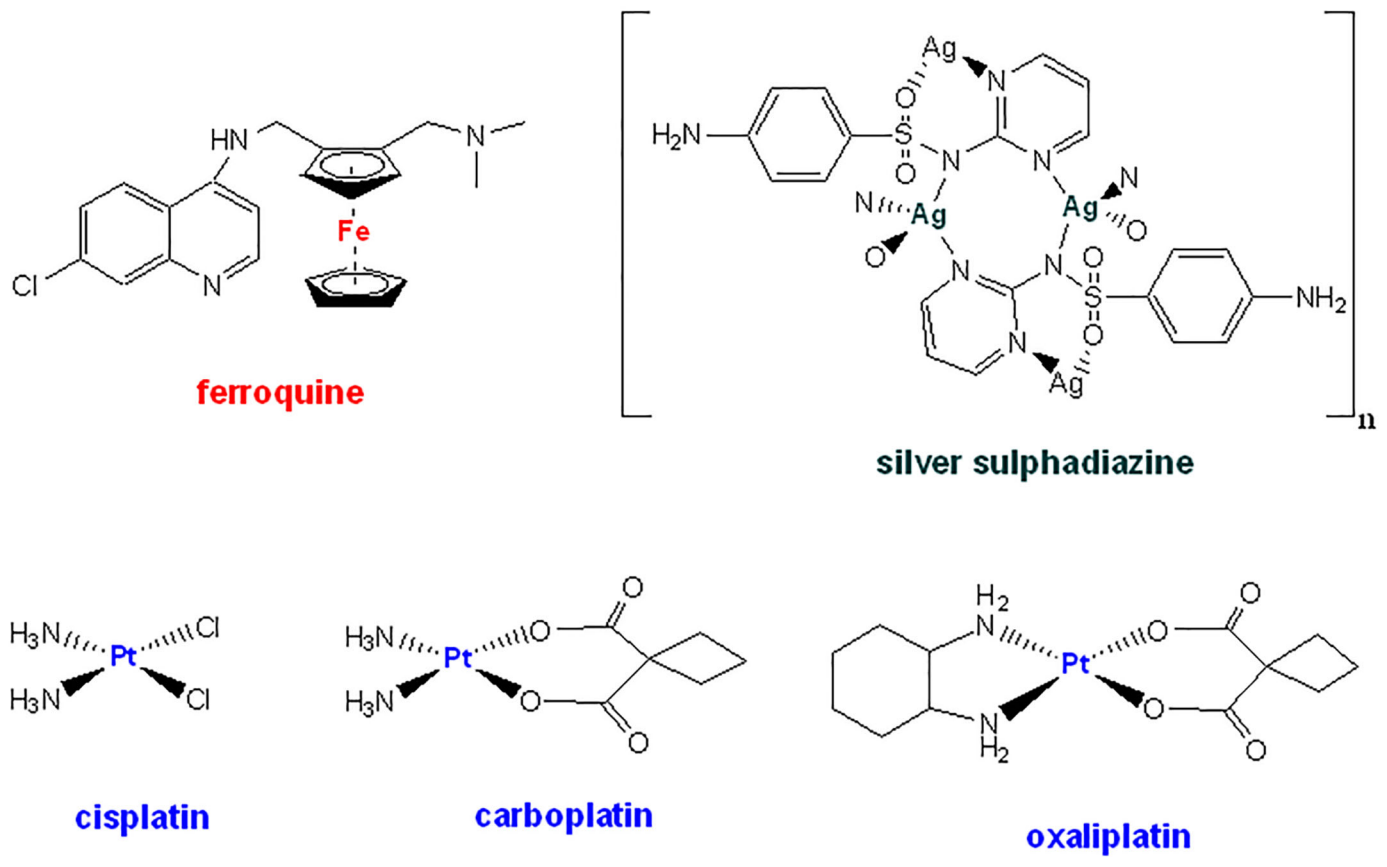
Chemical structures for the most relevant metallodrugs with antimicrobial properties.

In addition, biofilms are groupings of microorganisms enveloped by a protective glycocalyx matrix made up of self-synthesized extracellular polymeric substances (EPS) that adhere to each other and can adhere to living or inert surfaces (Mohammed et al., 2018; Aguilera-Correa et al., 2021). They bring many advantages to microorganisms, including increased resistance to extreme environmental conditions, energy storage, resistance to antimicrobial agents (antibiotics, reactive oxygen species, and heavy metals), and protection against host immune response such as phagocytosis (Flemming and Wingender, 2010). Moreover, the structure of biofilms can be modified giving the bacterium the flexibility to adapt quickly to different conditions (Hall-Stoodley et al., 2004). In this regard, staphylococci are known to be good biofilm formers, especially *S. aureus* (Otto, 2012). Bacterial resistance is a serious health problem on a global scale, as well as an important economic challenge. It is estimated that around 33,000 people die every year from hospital infections caused by resistant germs assuming a cost of 1,500 million euros per year in Europe (Dadgostar, 2019). Thus, the main problems of resistance at hospitals are caused by Gram-positive bacteria including methicillin-resistant *S. aureus* (MRSA) and at the community by *Streptococcus pneumoniae* resistant to penicillin and macrolides. Unfortunately, far from disappearing, these problems persist today with prevalence around 25-30% of the total of both pathogens (Magiorakos et al., 2012). In addition, a small number of antibiotics have been approved in recent years, the vast majority not being new drugs, but derived from existing families of antibiotics with already known resistances (Maglangit et al., 2021). In this context and due to the large history of metal compounds with antimicrobial properties, metallodrugs based on iron, cobalt, copper or silver have been widely evaluated (see chemical structures of relevant metallodrugs with antimicrobial properties in **Figure 1**) (Harrison et al., 2008; Chang et al., 2010; Sim et al., 2018). Topical silver sulphonamide is applied to prevent and treat superficial burns (Aziz and Abdul Rasool Hassan, 2017). The antimalaria agent ferroquine (NCT03660839) and the antifungal agent VT1161 (NCT03561701) are currently in clinical trials (Wani et al., 2015; Brand et al., 2021). The most relevant platinum metallodrug correspond to the best-know cisplatin, developed by Rosenberg and co-workers in the 1960s (Ndagi et al., 2017). Modification of its structure gave rise to other platinum entities which some of them are successful therapeutic metallodrugs even today, such as carboplatin or oxaliplatin. However, platinum derivatives have not been so fruitful in the field of antimicrobial and antiparasitic metallodrugs. In fact, scarce scientific literature related to antibacterial effects of platinum derivatives exist mainly because the effective control of infections by antibiotics and the more urgent need for new anticancer treatments. Recently, the antimicrobial effects of platinum(II) complexes containing 1,10-Phenanthroline, and platinum(II) cyclooctadiene complexes represent promising alternative based on platinum for controlling resistant strains of *C. jejuni* and MRSA, respectively (Ravera et al., 2018).

The development of novel antibacterial metallodrugs aims at improving the antimicrobial activity of an existing drug by binding the drug (organic ligand) or organic compounds to a metal (Abdelhamid and Mathew, 2022). Thus, the coordination of the metal to the drug may offer a multimodal and metal-specific mode of action against antimicrobial resistance, which differs from purely organic drugs (Ravera et al., 2018). Herein, a novel metallodrug (2P-Pt) based on platinum and the successful previous results reported for the ligand 1,1’-(2-(4-isopropylphenyl) ethane-1,1-diyl) bis(3,5-dimethyl-1H-pyrazole) (2P) (Seguí et al., 2021; Ocaña et al., 2022) has been synthesized, its antibacterial effect against the planktonic and biofilm state of MRSA were evaluated and its mechanism of action was studied.

## Material and methods

### Synthesis of the novel metallodrug 2P-Pt

The PtCl_2_(DMSO)_2_ were synthesized following the same procedure as previously described (Andrade and Martins, 2019). ^1^H NMR (400 MHz), ^13^C-{^1^H}-NMR (101 MHz) and ^195^Pt NMR (64 MHz) spectra were recorded on a Bruker spectrometer at 297K by dissolving 2P-Pt in CDCl_3_. The ^1^H- and ^13^C-{^1^H}-NMR chemical shifts (δ) were expressed as ppm in relation to TMS and ^195^Pt NMR to K_2_PtCl_6_. Coupling constants (*J*) were documented in Hz. The IR experiments were conducted on FT/IR-4000 Series Jasco Instruments. The UV–Vis absorption spectra were recorded at room temperature by a Cary 100 (Varian) spectrophotometer using a slit width of 0.4 nm and a scan rate of 600 nm/min. Elemental Analysis was performed using an Elementary Chemical Analyzer LECO CHNS-932.

2P compound was obtained according to procedures reported in the literature (Seguí et al., 2021). The synthesis of 2P-Pt was performed by dissolving PtCl_2_(DMSO)_2_ (0,24 mmol, 100mg) in 10 mL of dichloromethane, and adding the 2P ligand (0,24 mmol, 80 mg). This mixture was refluxed overnight, turning the solution from colorless to yellow. The solvent was removed under vacuum and the resulting product washed with 5 mL of diethyl ether. The 2P-Pt complex was obtained as a yellowish power in high yields (Andrade and Martins, 2019).

Yield: 107mg, 0.178mmol, 75%. ^1^H NMR (400 MHz, CDCl_3_) δ 7.09 (m, *J* = 8.3 Hz, 4H, cym), 5.95 (t, *J* = 7.9 Hz, 1H, C*H*-CH_2_), 5.84 (s, 2H, H_4_), 5.36 (d, *J* = 7.9 Hz, 2H, CH-C*H_2_*), 2.87 – 2.75 (m, *J* = 13.5, 6.7 Hz, 1H, CH_3_-C*H*-CH_3_), 2.55 (s, 6H, Me^3^), 1.98 (s, 6H, Me^5^), 1.16 (d, *J* = 6.9 Hz, 6H, C*H_3_*-CH-C*H_3_*). ^13^C-{^1^H}-NMR (101 MHz, CDCl_3_) δ 154.0 (2C, pyrazol quaternary), 148.9 (1C, cym quaternary), 141.0 (2C, pyrazol quaternary), 131.1 (1C, cym quaternary), 129.5 (2C, cym), 127.1 (2C, cym), 108.1, 110.2 (2C, C_4_), 70.4 (1C, *C*H-CH_2_), 41.7 (1C, CH-*C*H_2_), 33.7 (1C, CH_3_-*C*H-CH_3_), 24.0 (2C, *C*H_3_-CH-*C*H_3_), 15.0 (2C, Me^3^), 11.1 (2C, Me^5^). ^195^Pt NMR (64 MHz, CDCl_3_) δ: -2112.80 ppm. UV-vis: maximum absorbance at 212 nm. IR: 2962-2921 cm^−1^ (C–H sp^3^ stretching), 1461–1394 cm^−1^ (two bands C=C aromatic stretching), 1416 cm^−1^ (C-H methyl group bending), 1021 cm^−1^ (C–N stretching). UV-vis: maximum absorbance at 212 nm.Elemental analysis calcd (%) for C_21_H_28_Cl_2_N_4_Pt: C, 41.87; H, 4.68; N, 9.30; found: C, 41.93; H, 4.45; N, 9.97.

### Antimicrobial activity studies

#### Clinical MRSA isolates

Three different strains of *S. aureus* were studied. A strain of *S. aureus* subsp. *aureus* from the American Type Culture Collection (ATCC) 25923 which is sensitive to various antibiotics, including methicillin, and is commonly used as a control strain for standard laboratory testing (Treangen et al., 2014), and two clinical MRSA isolated in the Department of Microbiology of the Jiménez Díaz Foundation University Hospital: MRSA1, isolated from the infected wound of a 73-year-old patient and MRSA2, isolated from the paronychia of a 92-year-old patient. All strains were conserved at -80°C until they were used with at least 24 h of culture in Tryptic Soy Agar supplemented with 5% sheep blood (TSS, Biomérieux, France) before each experiment.

#### Biofilm forming capacity

The biofilm forming capacity of the different strains was assessed according to a previously published and widely accepted protocol (Seguí et al., 2021). The colonies of each of the strains cultivated in TSS agar plates, were suspended in Tryptic Soy Broth, (TSB) until reaching a turbidity comparable to 0.5 on the McFarland scale (∼1.6×^8^ colony-forming units per milliliter, CFU/mL). This suspension was diluted 100 times in TSB supplemented with 1% glucose (Sigma Aldrich, United States) to reach a bacterial concentration of approximately 10^6^ CFU/mL. Afterwards, 200μL of the cell suspension was added in 24 of the 96-well flat-bottomed plate (MicroWell, Thermo Fisher Scientific, United States). The plate was incubated for 24 h at 37°C in 5% CO_2_. The supernatant was removed, and each well was washed twice with 200 μL of 0.9% NaCl saline (B. Braun, Germany). After that, the remaining adhered bacteria biofilm were fixed with 200 μL of absolute methanol (PanReac, Spain) for 20 min. The methanol was discarded, and the plates were dried at room temperature. Finally, the biofilm formed was stained with 150μL of 2% violet crystal for 15 min. After staining, each well was washed twice with 200μL of distilled water. After washing, the violet crystal was solubilized with absolute ethanol (PanReac, Spain) for at least 5 min. Absorbance was measured at a wavelength of 570 nm. The optical density control (ODc) was defined as the mean of negative control (wells only with culture medium and without bacteria) plus three times its standard deviations. The strains were classified according to their OD per well within the following categories: non-biofilm former (0 ≤ $\frac{OD}{ODc}$ ≤ 1), weak biofilm former (1 ≤ $\frac{OD}{ODc}$ ≤ 2), moderate biofilm former (2 ≤ $\frac{OD}{ODc}$ ≤ 4) and strong biofilm former ( $\frac{OD}{ODc}$ ≤ 4) (Stepanović et al., 2007).

#### MIC (minimum inhibitory concentration) and MBC (minimum bactericidal concentration) determination on planktonic bacteria

The MIC was determined using the broth microdilution method from a 0.5 McFarland bacterial suspension previously prepared in saline serum for each strain. MIC is defined as the minimum concentration needed to inhibit the growth of a microorganism. First, a series of double dilutions of 2P-Pt at concentrations of 1,000 to 0.965625 mg/L were added to the Müeller-Hinton cation-adjusted broth (CAMHB) (Sigma Aldrich, St. Louis, MO, USA). USA United States) up to a final volume of 100 μL per well on a 96-well round-bottom polypropylene plate (Corning Inc., Corning, USA). One hundred microliter of the bacterial suspension in CAMHB was added, in a 1:100 dilution, containing approximately 1.6×10^6^ CFU/mL. A static 24-h incubation at 37°C in 5% CO_2_ was followed. After incubation, bacterial viability was determined by adding 20 μl of 5 mg/mL of 3-(4,5-dimethylthiazole-2-yl)-2,5-diphenyltetrazol (MTT) per well (Sigma Aldrich, Merck, Darmstadt, Germany) and incubating at 37°C (100 rpm) for 1 h. Once incubation was completed, columns that did not have any color change were rated as higher than the MIC value and absorbance was measured at a wavelength of 570nm (Aguilera-Correa et al., 2022a). The MBC was determined with the methodology Flash microbiocide using the previously MIC plate (Hernandes et al., 2023). MBC is defined as the minimum concentration needed to kill a specific bacterial concentration. After 24-hour incubation of the MIC plate, 20 μL from each well was transferred to a new 96-well flat-bottom plate adding 180 μL of TSB. A 24-h static incubation at 37°C in 5% CO_2_ was followed. After incubation, the MBC was determined by measuring absorbance using a wavelength of 600 nm (Aguilera-Correa et al., 2022b). These experiments were performed four times.

#### MBIC (minimal biofilm Inhibitory Concentration) and MBEC (minimal biofilm Eradication Concentration) determination on biofilm forming bacteria

The MBIC and the MBEC were determined using the methodology described above. MBIC is the minimum concentration needed to inhibit the visible growth of a bacterial biofilm. For the MBIC, biofilm formation was induced at the bottom of a 96-well flat-bottom plate (Thermo Fisher Scientific, Waltham, MA, USA) by inoculating 100 μL of CAMHB with 10^5^ CFU/mL of bacteria per well. A 24-h incubation at 37°C in 5% CO_2_ was followed. The supernatant was aspirated. 200 μL of the 2P-Pt material was deposited per well with different concentrations ranging from 2,000 to 125 mg/L. The plate was incubated at 37°C in 5% CO_2_ for at least 20 h. After incubation, the MBIC was determined using the MTT assay, as described above, and by measuring absorbance at 570 nm. MBEC is the minimum concentration needed to kill the bacterial biofilm. For MBEC, the biofilm from the bottom of each well was scraped and mixed with the supernatant using sterile 200 μL tips. After that, 20 μL of each well was transferred to a new 96-well plate with 180 μL of TSB broth per well. A 24-h static incubation at 37°C in 5% CO_2_ was followed. After incubation, the MBEC was determined by measuring absorbance with a wavelength of 600 nm (Mattie et al., 1989). These experiments were performed four times.

#### Effect on the development of biofilm in a wound-like medium

The development of biofilm in a wound-like medium (WLM) was determined as previously described (Aguilera-Correa et al., 2021). Briefly, the WLM is composed of 45% Bolton broth (Sigma-Aldrich), 50% adult bovine serum (Sigma-Aldrich), 5% lacquered horse blood (Thermo Fisher Scientific), and with or without (positive control) 2P-Pt at a concentration of 10 mg/L. Two hundred fifty microliter of each medium with 2 μL of 10^8^ CFU/mL of each strain were incubated at 37°C in 5% CO_2_ in sterile Eppendorf tubes (2 mL) for 24h. After incubation, 750 μL of saline solution were added and each Eppendorf tube was sonicated for 5 min using a low-power bath sonicator (50-60 Hz) (Ultrasons-H 3,000,840, J. P. Selecta, Abrera, Spain). This sonicated saline solution was serially diluted with saline solution. UFC/mL was estimated using the drop plate method on TSS agar plates (Aguilera-Correa et al., 2021).

### Antibacterial mechanism studies

#### Cation-mediated: Determination of MIC and MBC with EDTA

Ethylenediaminetetra-acetic acid (EDTA) is a chelating agent with the ability to capture ions from various metals (Finnegan and Percival, 2015). The MIC and MBC with EDTA was only performed for *S. aureus* ATCC 25923 using the broth microdilution method, applying 2P-Pt dilutions in CAMHB at concentrations of 1,000 to 0.965625 mg/L. The final concentration of EDTA for treatment was 50 μM and the inoculum of bacterial strains was 1.6×10^6^ CFU/mL (Raad et al., 2008). To determine the values of MIC and MBC, the same procedure described previously was followed in this paper.

#### Oxidative stress mediated: Determination of MIC and MBC with DMSO

The toxicity of the different compounds used in the treatment of bacterial infections can cause oxidative stress in the bacterial cell by generating reactive oxygen species (ROS). Accumulation of ROS causes the oxidation of essential macromolecules of the cell that induces the cell death. The determination of oxidative stress is usually interesting to observe if this is the main reason for the antibacterial activity of the different compounds (Díaz-García et al., 2019). Dimethyl sulfoxide (DMSO) is a compound with the capacity of capturing ROS, specifically the hydroxyl radicals, neutralizing their bactericidal activity (Djurišić et al., 2015). To determine ROS-mediated antibacterial activity in ATCC 25923, 2% DMSO was added to the treatment as the final concentration, using dilutions of 2P-Pt at concentrations of 500 to 31.25 mg/L. The values of MIC and MBC were obtained following the same protocol previously described in this work.

### Transmission electron microscopy studies

To visually support the numerical results obtained in the MIC and MBC assays, the experiment was analyzed using transmission electron microscopy (TEM) in ATCC 25923, described above. Resin polymerized bacteria were cut in semi-thin sections (0.6 μm) for optical microscopy and in thin sections (60 nm) for Transmission Electron Microscopy (TEM) using a Leica Ultracut UC7 ultramicrotom (Leica Wetzlar, Germany). The sections were collected in 200-mesh nickel grids and examined with a JEOL JEM 1400 transmission electron microscope (Jeol Ltd., Tokyo, Japan) (Aguilera-Correa et al., 2022b).

### Statistical analysis

The statistical analysis was performed using GraphPad prism 9.1.2 software (United States). A statistical significance of 0.05 was used. The statistical significance between the different groups was determined using the non-parametric Kruskal-Wallis test, for more than two groups, and the non-parametric Mann-Whitney test, for the comparison of two groups. Results are represented as median and interquartile range.

## Results

### Synthesis and characterization of 2P-Pt

**Figure 2:**
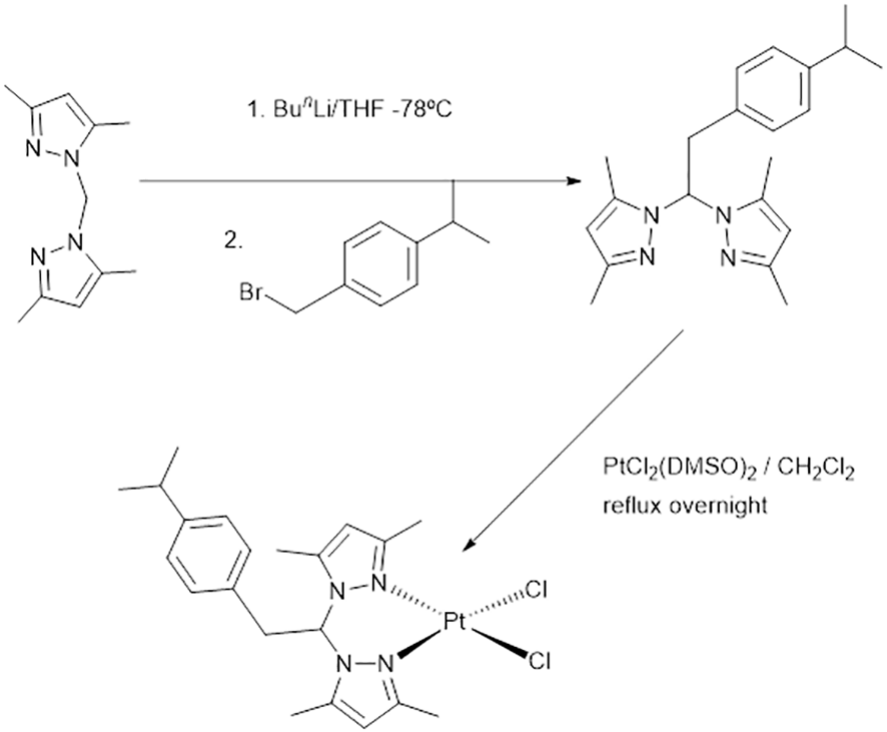
Synthesis of the platinum metallodrug 2P-Pt.

The synthesis of the platinum compounds 2P-Pt followed the scheme depicted in **Figure 2**. The metal precursor cis-[Pt (DMSO)_2_Cl_2_] was reacted with 1 molar equivalent of the ligand 2P in dichloromethane and the product obtained as a yellowish solid in high yields. The complex is air stable in the solid state and soluble in organic solvents such as DMSO, methanol or dichloromethane.

**Figure 3:**
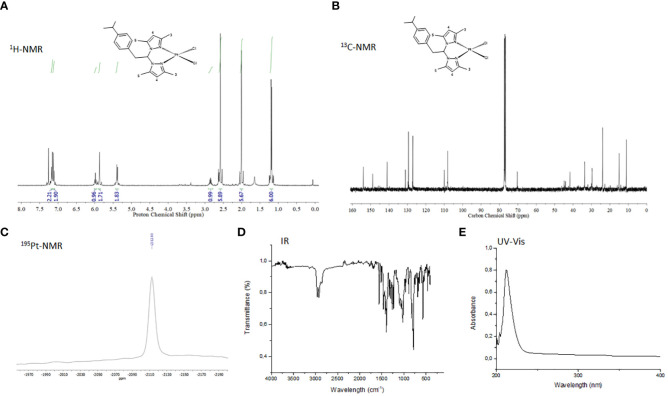
**(A)** ^1^H NMR spectrum of 2P-Pt in CDCl_3_ at 297(K) **(B)** ^1^H-{^13^C} NMR spectrum 2P-Pt in CDCl_3_ at 297(K) **(C)** ^195^Pt NMR spectrum of 2P-Pt in CDCl_3_ at 297K. **(D)** IR spectrum of 2P-Pt. **(E)** UV spectrum of 2P-Pt in CH_2_Cl_2_. **(F)** Selected ^1^H spectra at different time of 2P-Pt in DMSO-d^6^:D_2_O/1:3.

2P-Pt was characterized by analytical methods, infrared (IR), ultraviolet-visible (UV-Vis), and nuclear magnetic resonance (NMR) spectroscopy. The NMR characteristics of the complex is consistent with those reported for related Pt(II) complexes (Pazderski et al., 2009). Structural elucidation is depicted in the experimental section. Signals in the ^1^H and ^13^C NMR spectra (**Figure 3**) were observed with the expected chemical shifts and assignment was performed based on 2D-^1^H ^13^C HSQC spectra. The presence of a multiplet around 7.1 ppm and at 125–150 ppm in the ^1^H and ^13^C{^1^H} NMR spectra, respectively, confirmed the presence of the *p*-cymene moiety. The pyrazol rings exhibit one set of resonance for the protons and carbons H^4^, Me^3^ and Me^5^, indicating that the pyrazole rings are equivalent (**Figure 3**). The results are consistent with a square plane geometry for the platinum centre, with the ligand coordinated in a *κ* ^2^-*NN* bidentate fashion. ^195^NMR spectrum of 2P-Pt revealed one signal around -2300 ppm which correspond to the region expected for the coordination of two chlorine and two nitrogen atoms to the metal. The IR spectra of 2P-Pt exhibited C–N stretching bands in the range of 1000 cm^−1^ attributed to the pyrazol rings and it was identified new bands at around 1461–1394 cm^−1^ due to the C=C aromatic stretching vibrations of the *p*-cymene moiety (Michelin et al., 2011). The 3000 cm^−1^ region showed absorptions attributed to C\H stretching of CH_2_ and CH_3_. The UV-Vis absorption spectrum of 2P-Pt was recorded in a DMSO solution (10^-5^ M) at 25°C. The absorption maximum of the 2P ligand moves hyperchromically to 212 nm after metalation. This strong band may refer to ligand-to-metal charge-transfer transitions between π-orbitals on Cl to d-orbitals of platinum. The stability of the new compound was tested in CDCl_3_ by ^1^H NMR spectroscopy and the compound were unchanged after three days in solution at room temperature. As it happens to many metallodrugs, the platinum compounds needed to be dissolved in a mixture of H_2_O:DMSO to perform biological assays which in any case did not exceed 0.25% v/v of DMSO. Therefore, the stability of the 2P-Pt in CDCl_3_ and DMSO-d^6^:D_2_O was carried out by NMR monitoring. The set of signals belonging to the starting complex persist throughout the stability experiments carried out in both experiments. The existence of only one pattern for 2P-Pt suggests ruling out fast chloride dissociation.

### Antimicrobial activity studies

#### MIC, MBC, MBIC and MBEC determination on planktonic bacteria

The antibacterial and antibiofilm effect of 2P-Pt in three different strains of planktonic bacteria expressed as MIC, MBC, MBIC and MBEC are reflected in **Table 1**.

**Table 1:** Minimum inhibitory concentration (MIC), minimum bactericidal concentration (MBC), minimum biofilm inhibitory concentration (MBEC), minimum biofilm eradication concentration (MBEC) of the different strains used in this study.

	*S. aureus* ATC25923	MRSA1	MRSA2
MIC (mg/L)	250	250	125
MBC (mg/L)	250	250	250
MBIC (mg/L)	500	500	500
MBEC (mg/L)	>2000	1000	>2000

#### Biofilm forming capacity

The three bacterial strains are strong biofilm formers: 45.4 (26.9-53.5) × OD_C_ for ATCC25923, 40.1 (31.1-47.5) × OD_C_ for MRSA 1, and 28.3 (26.1-31.1) × OD_C_ for MRSA 2. The OD/ODc value from all of them was ≥ 4.

#### Effect on the development of biofilm in wound-like medium

**Figure 4:**
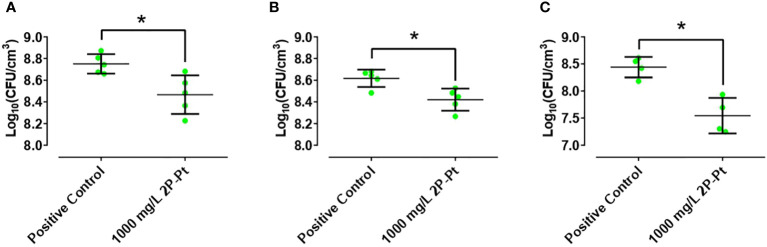
Effect of 2P-Pt on the development of biofilm in a wound-like medium at a concentration of 1,000 mg/L in ATCC 25923 **(A)**, MRSA 1 **(B)** and MRSA 2 **(C)**. *p-value< 0.05 in Wilcoxon test. The bars represent the interquartile range.

The antibiofilm activity of 2P-Pt in a wound-like medium was evaluated in the three strains: *S. aureus*: ATCC 25923, MRSA 1 and MRSA 2, at a concentration of 1,000 mg/L. The compound 2P-Pt decreased the development of biofilm in comparison to the control in 44.9% (**Figure 4A**), 36,7% (**Figure 4B**) and 89.7% (**Figure 4C**) for the strains ATCC25923, MRSA 1 and MRSA 2, respectively.

### Antibacterial mechanism studies

#### Cation-mediated antibacterial mechanism: Determination of MIC and MBC with EDTA

EDTA influence on the antibacterial effect of 2P-Pt was evaluated by studying the MICs and MBCs of different concentrations in the ATCC 25923 strain of *S. aureus*. In the control group without EDTA and in the group with 50 μM EDTA, the values of MIC and MBC were 125 mg/L and 250 mg/L, respectively.

#### Oxidative stress-mediated antibacterial mechanism: determination of MIC and MBC with DMSO

The presence of exogenous ROS, induced by the 2P-Pt complex as an antibacterial mechanism, was evaluated by studying the MICs and MBCs in the ATCC 25923 strain of *S. aureus*. In the control group without DMSO and in the group treated with a concentration of 2% DMSO, the values of MIC and MBC were 125 and 250 mg/L, respectively. The similarity of these values demonstrates that the bactericidal effect of the 2P-Pt complex is not mediated by exogenous ROS production.

#### Transmission electron microscopy studies

**Figure 5:**
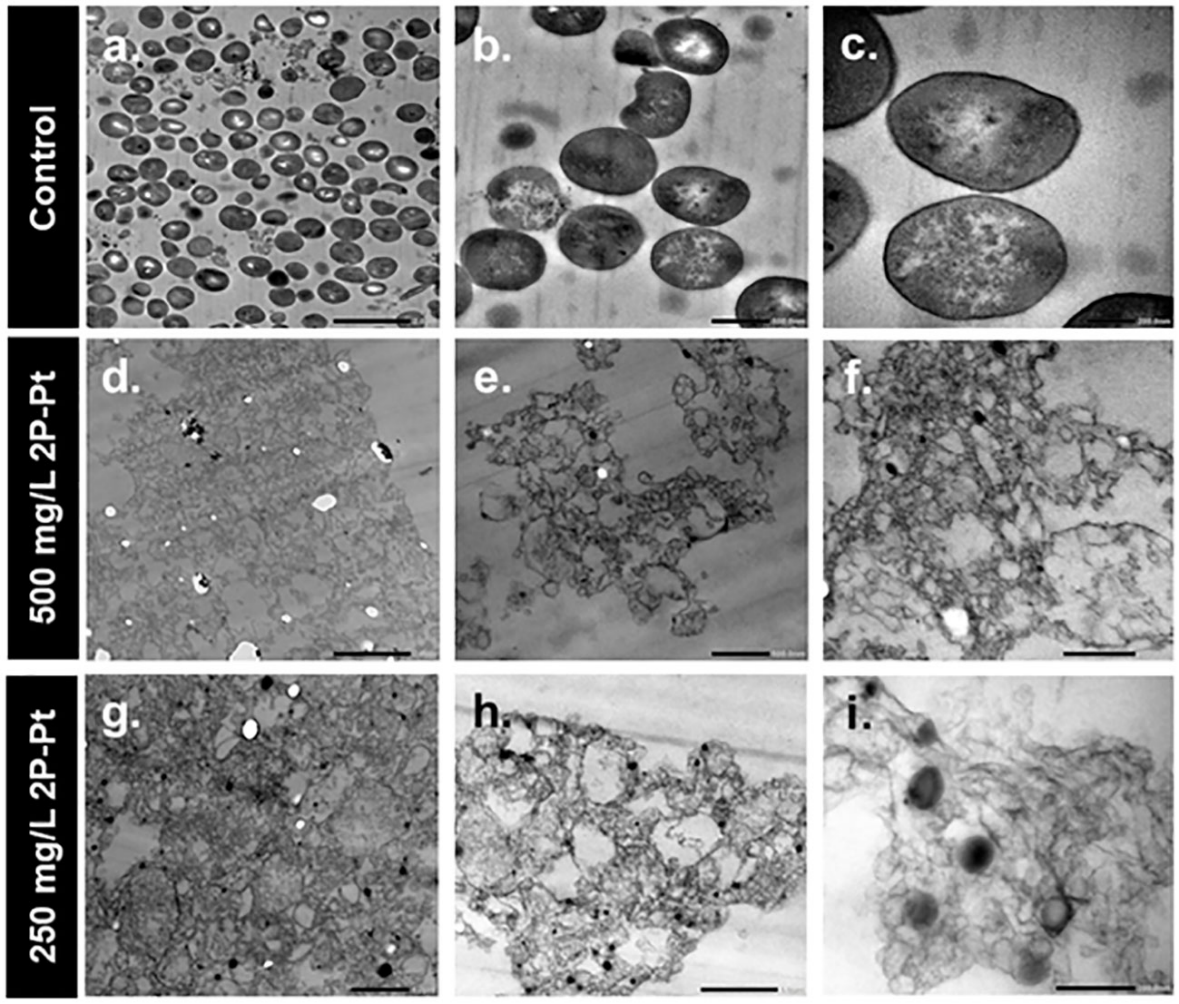
TEM images of planktonic cells of S. aureus ATCC 25923 versus control **(A–C)** and treated with the 2P-Pt complex at concentrations of 500mg/L **(D–F)** and 250 mg/L **(G–I)**.

The results of the antibacterial activity of 2P-Pt observed in the MIC and MBC experiments were confirmed using TEM, analyzing both the sensitive strain (ATCC 25923) and the MRSA clinical strains (MRSA 1 and MRSA 2) (**Figure 5**). The TEM technique allowed the visually inspection of the morphological changes induced by 2P-Pt in these bacteria. In the control group, cocci with normal morphology and an intact cell wall were observed (**Figure 5A**). On the other hand, the treated group, treated with different concentrations of 2P-Pt, bacterial debris were identified because of cell lysis at both 500 mg/L (**Figure 5D–F**) and 250 mg/L (**Figure 5G–I**). Moreover, in some cases vacuoles could be observed representing spherical membrane debris (**Figure 5**).

## Discussion

*S. aureus* is a type of bacteria found on human skin and can colonize distinct parts of the body such as the nostrils, vagina, urethra, and gastrointestinal tract (Lowy, 1998; Harris et al., 2002). It is estimated that up to 20% of the healthy adult population is colonized by this microorganism asymptomatically (Lowy, 1998). However, *S. aureus* infection can progress to a serious illness in immunocompromised persons as well as in patients with other pathologies like diabetes, chronic kidney disease, or cancer (Frost et al., 2019; Cheung et al., 2021). Moreover, it is very well-known that *S. aureus* can cause a wide range of clinical infections such as endocarditis, gastroenteritis, meningitis, toxic shock syndrome, and urinary tract infections, among others (Tong et al., 2015). Treatment regarding *S. aureus* infection by antibiotics generates resistance which limit successful clinical outcomes for patients (Pantosti et al., 2007). In fact, MRSA is considered a pandemic pathogen (Craft et al., 2019), being responsible of up to 76% of skin and soft tissue infections (Parlet et al., 2019). Accordingly, there is clearly an unmet clinical need for new treatments able to overcome resistance in the treatment of MRSA. In this regard, even though platinum derivatives have been mainly studied as antitumor compounds, their antibacterial properties are well documented. Some examples of platinum compounds with antimicrobial activity are the platinum cyclooctadiene complexes effective against a wide variety of Gram-positive species or those containing 1,10-phenantroline in their coordination sphere for controlling resistant strains of *C. jejuni* or *E. coli* (Harrison et al., 2008; Ng et al., 2013; Lacerda et al., 2022).

In a previous study, we reported the inhibitory capacity of the organic ligand 2P against Gram-positive bacteria such as *S. aureus* and *E. faecalis* (Seguí et al., 2021) Despite showing very low cytotoxicity (Ocaña et al., 2022), the no eradication of the bacteria in their planktonic state might hamper its clinical use (Seguí et al., 2021). 2P was designed to contain a *p*-cymene moiety within its structure. Such moiety is an aromatic monoterpene of the alkyl group that is naturally found in many species of plants, including cumin and thyme, and is currently used in food chemistry and medicine for its wide therapeutic properties (Balahbib et al., 2021). It was ascertained that such moiety in the ligand structure played a significant role in the efficiency observed for this family of ligands. Bearing in mind the inhibitory effect of this organic ligand, we hypothesized that the combination of 2P and platinum, a metal of interest because of antibacterial, antiviral, and anticancer properties (Mbaba et al., 2020; Rottenberg et al., 2021; Abdelhamid and Mathew, 2022), by the synthesis of a novel platinum metallodrug (2P-Pt) could show clinical benefits for overcoming resistance in the treatment of MRSA. The synthesis of the novel metallodrug 2P-Pt was straightforward (**Figure 2**). The metal precursor cis-[Pt (DMSO)_2_Cl_2_] was reacted with 1M equivalent of 2P in dichloromethane to give rise to 2P-Pt in a very good yield. The metallodrug was fully characterized by analytical and spectroscopic methods (**Figure 3**). 2P-Pt showed the ability of inhibiting the growth and eradicating all MRSA strains evaluated in the planktonic state. MIC and MBC for ATCC 25923, MRSA 1 and MRSA 2 that support this assertion. For instance, the MIC and MBC values of 2P for *S. aureus* ATCC25923 were 62.5 and 2000 µg/mL (Seguí et al., 2021), respectively, whilst the MIC and MBC values of 2P-Pt for *S. aureus* ATCC25923 were 250 and 250 µg/mL, respectively, which means that 2P-Pt showed a bactericidal effect at a lesser 8-fold concentration (250 mg/L) than 2P (2000 mg/L). These results show the preclinical benefit that the combination of 2P and platinum could have for the treatment of MRSA. Likewise, other studies have also showed that the metal complexes have a greater antimicrobial activity than the free ligands (Frei et al., 2020).

In relation to the MRSA biofilms, we have shown that the 2P-Pt complex was also able to inhibit its growth, but not to eradicate it. In addition, 2P-Pt was able to significantly decrease the development of the biofilm in a wound-like medium (**Figure 4**), a method that provides a more realistic *in vitro* biofilm model by simulating some of the functional characteristics of chronic pathogenic biofilms under *in vivo* conditions (Aguilera-Correa et al., 2021). Bacterial biofilms are very challenging to treat with available antibiotics because they are not capable of interacting with the deeper parts of the biofilm. In fact, there is a need to develop novel antimicrobial agents that can significantly inhibit biofilm formation. Novel compounds such as 2P-Pt with efficient bactericidal effects and antibiofilm effects could come up as potential alternative and complementary agents against biofilm associated microbial infections. The incapacity of 2P-Pt to eradicate MRSA biofilm may be explain on the basis that biofilms become between 10 and 1,000 times more resistant to multiple antimicrobial compounds (including antibiotics) when comparing to the same bacterium in planktonic form (Davies, 2003). It has been suggested that the structure of the EPS matrix, the reduced metabolic rate and the way bacteria grow in the biofilm, especially those found in the deeper layers of the biofilm, could be some of the important factors in making them intrinsically resistant to high concentrations of antimicrobials of different nature (Davies, 2003).

In our previous work, we proposed that 2P antimicrobial properties were probably based on several factors, including high pH and osmotic effects caused by the non-physiological concentration of dissolved ions, as observed for other compounds (Marchese et al., 2017; Seguí et al., 2021). However, platinum is likely to undergo ligand exchange reactions when exposed to solvents and medium (Frei et al., 2020), and the release of platinum ions is the main mechanism reported for inducing the antimicrobial activity of these compounds (Godoy-Gallardo et al., 2021). In drug discovery, the understanding of the mechanism of action may subsequently aid the potentially rational design of improved drugs. In a first approach, we have studied possible action mechanisms of the 2P-Pt complex. Thus, elucidating whether the antimicrobial mechanism of the 2P-Pt complex was mediated by extracellular ROS generation, MIC and MBC trials were studied in presence and absence of an attenuator of ROS (Price et al., 2009), and the same values of MIC and MBC. Therefore, the antibacterial mechanism of 2P-Pt seems not to be mediated by the generation of extracellular ROS, contrary to what many authors have previously reported (Alsalme et al., 2016; Mbaba et al., 2020). In addition, finding out if the mechanism of the antibacterial effect of 2P-Pt was mediated by the extracellular release of platinum cations from the 2P-Pt complex in the presence of the bacterium, the MIC and MBC assays were repeated in the presence of EDTA, a chelating agent of divalent metal cations (Ravalli et al., 2022). The results with and without EDTA were also very similar, and therefore, the antibacterial mechanism of 2P-Pt does not seem to be mediated by the extracellular release of divalent platinum cations. However, more studies are needed to ascertain the mechanism of action of these metal complexes.

Finally, the lysis capacity of the 2P-Pt complex against MRSA was confirmed by TEM studies, where bacterial remains were observed because of cell lysis (**Figure 5**). In this regard, according to our results, this lytic effect does not seem to be due to the extracellular effect of either exogenous ROS or platinum cations release, and thus, it might be hypothesized that this bacterial lysis is due to an intracellular effect of 2P-Pt. Different action mechanisms of 2P-Pt against bacterial growth are proposed but not fully understood yet, being still needed further studies to determine the antimicrobial mechanisms of 2P-Pt on both planktonic and biofilm forms of MRSA. However, Tweedy’s chelation theory states that chelation reduces the polarity of the metal ion due to the partial sharing of its positive charge with the donor group or ligand and the possible delocalisation of π-electrons throughout the chelate ring system formed during the formation of the complex. This process increases the lipophilic capacity of the metal atom and, consequently, the hydrophobic capacity and liposolubility of the complex, which would favour its permeability through the lipid membrane of the microorganism reaching its cytoplasm (Castillo-Blum and Barba-Behrens, 2000; Lunagariya et al., 2017; Gaber et al., 2018). Once inside the cytoplasm, Pt could dissociate from the 2P-Pt complex, with each compound exerting its own antibacterial effect. Thus, a recent study has shown that zinc, a divalent cation like Pt, can compromise *Streptococcus pneumoniae* peptidoglycan formation by intracellularly inhibiting the enzyme GlmU, an enzyme that catalyses the last two sequential reactions in the *de novo* biosynthetic pathway for UDP-N-acetylglucosamine (Brazel et al., 2022). Therefore, if the 2P-Pt complex were to decomplex in the cytoplasm of *S. aureus*, the released Pt could inhibit the GlmU enzyme, as zinc does, blocking peptidoglycan synthesis and causing the staphylococcal lysis, alone or in company with the possible intracellular antibacterial effect of 2P.

Our study shows two main limitations. First, the one related to the high cost of platinum since this metal is 10,000 times more expensive than other metals such as iron (Zhang et al., 2017) which would make large-scale production costly. However, platinum drugs have been the cornerstone of cancer therapies for ages and the cost of production has been overcome by successful outcomes in clinic. Second, the toxicity of platinum. Adverse effect coming from platinum therapy in cancer is very well known which always depend on the auxiliary ligand coordinated to the metal center. In the same way, platinosis as a chronic disease is reported during excessive exposition to the salts of the platinum group metals. Other harmful effects of Pt has been described in plants, rats and humans (Ravindra et al., 2004; Gagnon et al., 2006) when they are exposed to Pt over time. However, the low proportion of 2P-Pt in this treatment, its topical use, and the plausible biochelation coming from the skin microflora (mainly Gram-negative bacteria) (Gopal et al., 2013; Yousef et al., 2021) would hamper any possible chronic or acute systemic side effect in patients.

## Conclusions

In conclusion, our results support 2P-Pt complex as a promising therapeutic alternative for treating infections caused by MRSA. 2P-Pt showed the ability to actively inhibit and eradicate the growth of MRSA planktonic state and reduce the development of its biofilm, both in conventional biofilm-inducing media and in a more similar *in vivo* condition media like a wound-like medium. These results suggest that the metallodrug 2P-Pt could be used to prevent staphylococcal infections at local level. Further studies are needed to understand the main mechanism of action of 2P-Pt and to demonstrate the *in vivo* efficacy of this novel metallodrug.

## Data availability statement

The raw data supporting the conclusions of this article will be made available by the authors, without undue reservation.

## Author contributions

SHN-C: Conceptualization, Investigation, Writing–review and editing, ED-J: Investigation, Supervision, Writing–review and editing, SLT-V: Investigation, Conceptualization, Writing–review and editing, RP-T: Conceptualization, Writing–review and editing, NM-M: Investigation, Supervision, Writing–review and editing, RA-R: Conceptualization, Writing–review and editing, AL-S: Investigation, Supervision, Writing–review and editing, JE: Conceptualization, Writing–review and editing, RL: Conceptualization, Writing–review and editing, CA-M: Conceptualization, Writing–review and editing, PS: Conceptualization, Writing–review and editing, AO: Conceptualization, Writing–review and editing, AL: Conceptualization, Supervision, Writing–review and editing, JJA-C: Investigation, Supervision, Writing–review and editing, FCP-M: Conceptualization, Investigation, Supervision, Writing–review and editing, MM: Conceptualization, Supervision, Writing–review and editing. All authors contributed to the article and approved the submitted version.
